# Pneumomediastinum by acute gastric dilation after binge-eating: a case report and literature review

**DOI:** 10.3389/fmed.2026.1719127

**Published:** 2026-02-26

**Authors:** Xin Lu, Shigong Guo, Huadong Zhu, Yi Li

**Affiliations:** 1 Emergency Department, State Key Laboratory of Complex Severe and Rare Diseases, Peking Union Medical College Hospital, Chinese Academy of Medical Sciences and Peking Union Medical College, Beijing, China; 2 Department of Rehabilitation Medicine, Southmead Hospital, Bristol, United Kingdom

**Keywords:** binge-eating, emergency medicine, gastric dilation, gastroenterology, pneumomediastinum

## Abstract

Acute gastric dilation (AGD) is a rare but critical medical condition characterized by a rapid and massive expansion of the stomach. While AGD secondary to binge-eating has been documented in literature, cases involving rapid consumption of carbonated beverages leading to acute gastric distention with subsequent pneumomediastinum are rarely reported. We present a case of a 17-year-old male who developed AGD following competitive ingestion of hamburgers and carbonated beverages, subsequently complicated by subcutaneous and mediastinal emphysema. Despite immediate gastric decompression via a nasogastric tube, the patient developed hemodynamic instability and oliguria. An emergency exploratory laparotomy was performed, during which a total of 3.6 liters of gastric contents were aspirated. The patient recuperated gradually under supportive care. This case highlights the necessity of early gastric decompression in binge-eating patients with significant carbonated beverage consumption who develop abdominal symptoms. The onset of hemodynamic instability or oliguria mandates urgent surgical intervention.

## Introduction

Acute gastric dilation (AGD) is a rare but critical medical condition characterized by rapid and massive expansion of the stomach due to the accumulation of excessive amounts of fluids, gas, or food (1, 2). It is potentially a life-threatening emergency that can lead to severe complications such as gastric rupture, abdominal compartment syndrome and pneumomediastinum (1, 3–5). The etiology is multifactorial, often involving mechanical causes (e.g., pyloric stenosis, gastric cancer or superior mesenteric artery syndrome) and systemic conditions (e.g., electrolyte imbalance, diabetes mellitus or binge-eating) (2, 6). While AGD secondary to binge-eating has been documented in literature (5, 7, 8), cases involving rapid consumption of carbonated beverages leading to acute gastric distention with subsequent pneumomediastinum are rarely reported.

Herein, we present a case of a 17-year-old male who developed AGD following competitive ingestion of hamburgers and carbonated beverages, subsequently complicated by subcutaneous and mediastinal emphysema.

## Case presentation

**Figure 1:**
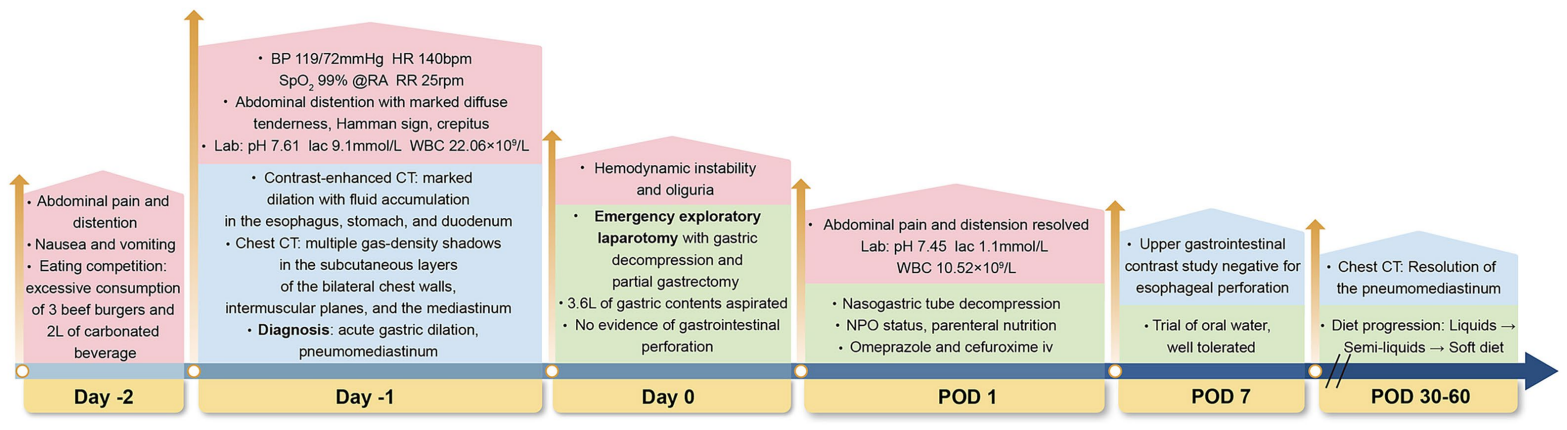
Timeline of diagnosis and management in a case of pneumomediastinum by acute gastric dilation after binge-eating. Day-2 (Symptom Onset); Day-1 (Hospital Admission); Day 0 (Emergency Surgery); POD, postoperative; BP, blood pressure; HR, heart rate; bpm, beats per minute; SpO_2_, oxygen saturation; RA, room air; RR, respiratory rate; rpm, respirations per minute; lac, lactate; WBC, white blood cell; CT, computed tomography pulmonary angiography; L, liter; iv, intravenous.

**Figure 2:**
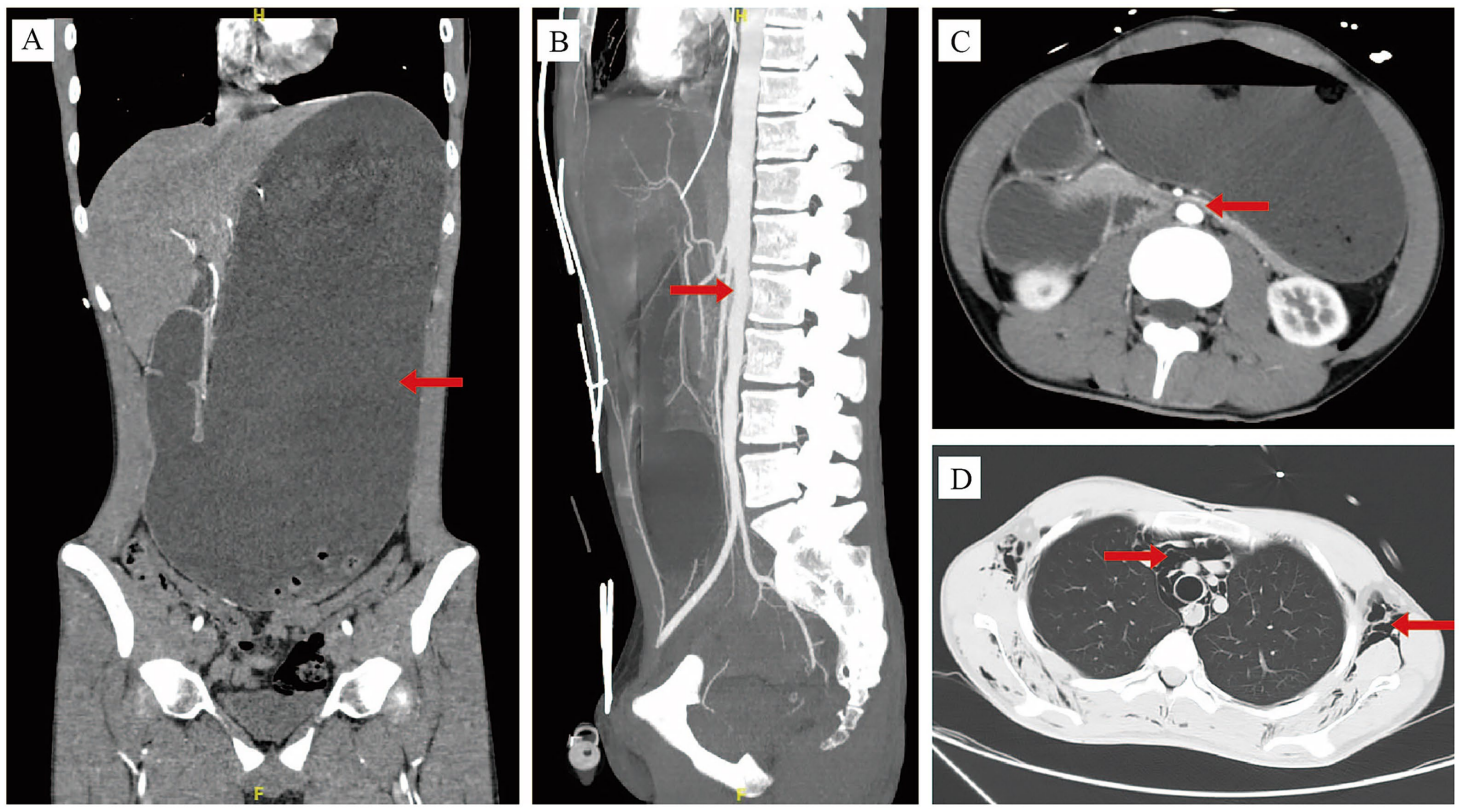
**(A)** Contrast-enhanced computed tomography showed massive gastric dilation. **(B)** The angle between the superior mesenteric artery and the abdominal aorta was approximately 15°. **(C)** Duodenal obstruction due to vascular compression. **(D)** Subcutaneous emphysema and pneumomediastinum.

A 17-year-old male (height: 187 cm; weight: 60 kg; body mass index: 17.1 kg/m^2^, classifying him as underweight) with no significant past medical history presented to the emergency department with a one-day history of abdominal pain and distention after excessive consumption of three beef burgers and two liters of carbonated beverage during an eating competition. Figure 1 outlined the timeline of diagnosis and management for this case. On admission, he had tachycardia (140 bpm), normal blood pressure (119/72 mmHg), tachypnoea (25/min), normal saturation (SpO_2_: 99% on air) and normal temperature (36.4 °C). Physical examination revealed abdominal distention with marked diffuse tenderness, most pronounced in the epigastric region, though no rebound tenderness or guarding was noted. Chest examination was significant for Hamman sign and crepitus. Arterial blood gas analysis revealed alkalosis with a pH of 7.61 and a lactate level of 9.1 mmol/L. Routine blood tests showed a raised white blood cell count of 22.06 × 10^9^/L, a hemoglobin level of 17.5 g/dL and a platelet count of 498 × 10^9^/L. Renal function tests showed that serum creatinine was 107 μmol/L and blood urea nitrogen was 11.3 mmol/L. Electrolyte levels revealed a low potassium concentration of 2.9 mmol/L and calcium level of 2.3 mmol/L. Contrast-enhanced computed tomography (CT) of the abdomen and pelvis demonstrated marked dilation with fluid accumulation in the esophagus, stomach, and duodenum, accompanied by air-fluid levels within the stomach (Figures 2A,C). In addition, the superior mesenteric artery (SMA) exacerbated the degree of duodenal obstruction, with a markedly reduced angle between the SMA and abdominal aorta measuring approximately 15° (Figure 2B). Notably, this radiological finding likely represented a secondary phenomenon due to massive gastric distension rather than primary superior mesenteric artery (Wilkie) syndrome, which is typically a chronic condition. Chest CT showed multiple gas-density shadows in the subcutaneous layers of the bilateral chest walls, intermuscular planes, and the mediastinum (Figure 2D). In response to radiological findings, AGD, subcutaneous emphysema and pneumomediastinum were diagnosed. Given the acute clinical context, the immediate priority was gastrointestinal decompression to address the life-threatening gastric dilatation, and a formal diagnosis of chronic SMA syndrome was not pursued.

The patient was admitted to the resuscitation room for emergency management. Despite immediate insertion of a nasogastric tube and aggressive fluid resuscitation, the patient developed hemodynamic instability and oliguria. The abdomen was firm with marked tenderness. Mottling was present on the lower extremities. The surgeon determined that there was an indication for emergency surgical gastrointestinal decompression. Although the patient had extensive subcutaneous and mediastinal emphysema, there was no significant mediastinal exudate or substantial pneumothorax/pleural effusion; therefore, surgical exploration of the esophagus was deferred. An esophagogram was planned post-decompression to rule out esophageal perforation. Subsequently, an emergency exploratory laparotomy with gastric decompression and partial gastrectomy were performed, during which a total of 3.6 L of gastric contents were aspirated. However, subsequent exploration of the abdominal cavity revealed no evidence of gastrointestinal perforation. Postoperatively, a nasogastric tube was placed for gastric decompression, and the patient was kept nil per os (NPO). Management included parenteral nutritional support, intravenous omeprazole for acid suppression, and intravenous cefuroxime for antibiotic prophylaxis. On postoperative day 1, laboratory studies revealed a white blood cell count of 10.52 × 10^9^/L, a hemoglobin level of 13.7 g/dL, and a platelet count of 249 × 10^9^/L. Arterial blood gas analysis showed a pH of 7.45 with a lactate level of 1.1 mmol/L. Serum electrolytes were within normal ranges, including potassium at 4.1 mmol/L and calcium at 2.12 mmol/L. An upper gastrointestinal contrast study performed on postoperative day 7 revealed no evidence of esophageal perforation. A trial of oral water was initiated and was well tolerated. The patient was discharged on day 7 post-surgery. Over the following month, the patient’s diet was progressively advanced from liquids to semi-liquids, and then to soft solids. At the two-month postoperative follow-up, a repeat chest CT scan demonstrated complete resolution of the pneumomediastinum.

Patient perspective: Looking back, this whole ordeal has really driven home for me how dangerous overeating can be. Being able to eat and drink by mouth again is something I’m truly grateful for and it feels like a huge relief.

## Discussion

AGD secondary to binge-eating represents a rare but serious complication, frequently associated with underlying eating disorders and psychiatric problems (6, 8–10). A structured review of the literature was performed to identify relevant cases of AGD following binge-eating. The search was conducted in PubMed for articles published from database inception to September 20, 2025. The search strategy employed a combination of key terms: (binge-eating OR binge) AND (“acute gastric dilation” OR “gastric dilation” OR “acute gastric dilatation” OR “gastric dilatation”). No language restrictions were initially applied. Search results were screened by title and abstract. Articles were included if they: (1) reported a clinical case of AGD; and (2) explicitly identified binge-eating or massive food ingestion as the primary precipitating factor. Studies where gastric dilation was due to other primary causes (e.g., intestinal obstruction, post-operative ileus) or animal studies were excluded. From the eligible cases, data on author and year of publication, patient demographics, past medical history and risk factors, specific binge foods or gastric contents, clinical presentation, and the method of gastric decompression employed were systematically extracted and compiled into a standardized summary table (Table 1). This table summarizes the cases of AGD associated with binge-eating. Notably, AGD has also been reported among individuals without prior medical history, occurring in contexts involving excessive food ingestion, such as celebratory events, food festivals, or refeeding after religious fasting (6, 7, 11). As demonstrated in our case, AGD can develop after competitive eating events. Diagnosis of AGD in previously healthy individuals following binge eating is often delayed due to the healthy individual’s higher physiological reserve, which in turn masks the severity of AGD.

**Table 1:** Summary of cases of acute gastric dilation associated with binge-eating.

No.	Author	Year	Gender	Age	Past history/Risk factors	Binge foods/Gastric contents	Clinical presentation	Gastric decompression
1	Amano (3)	2023	F	12	Carbohydrate-restricted diet	Grilled meat/2.865 L of solid and liquid contents	Abdominal pain, vomiting, mildly distended abdomen Pneumomediastinum	Nasogastric tube
2	Huang (6)	2023	F	14	Celebrate success with school exams	Binge eating	Severe abdominal pain Pneumatosis of the gastroduodenal wall and intrahepatic portal vein	Proximal subtotal gastrectomy
3	Tominaga (16)	2023	F	55	Eating disorder	Binge eating/5 L of food residue	Abdominal distension, rigidity Skin mottling	Nasogastric tube Upper endoscopy
4	Jano (17)	2022	F	28	Food festival	5 meals within 3 h/7 L of gastric contents	Diffuse abdominal pain, nausea, inability to vomit, and obstipation Rigid abdomen with evident peritonitis	Nasogastric tube Partial gastrectomy
5	Franzoi (1)	2023	F	41	Eating disorder	22 L of stomach contents	Progressively worsening abdominal pain, distended, signs of peritoneal irritation Shock, abdominal aorta was completely compressed	Total gastrectomy
6	John (7)	2023	M	20	Religious fast	Heavy dinner/much undigested food	Abdominal pain, rigid grossly distended silent abdomen Tachycardia, hypotension, anuria	Nasogastric tube Exploratory laparotomy
7	Han (5)	2022	F	21	Binge-eating order	Binge-eating, laxatives and sodium bicarbonate	Severe epigastric pain, vomiting, distended with generalized tenderness Legs were completely ischemic secondary to inferior vena cava occlusion	Needle decompression Laparotomy
8	Han (5)	2022	M	63	Gastro-esophageal reflux disease	A large Mexican dinner of nachos, a full bottle of red wine	Worsening epigastric pain, vomiting, tender with guarding Pneumoperitoneum	Laparotomy
9	Weinstein (11)	2022	F	28	Binge-eating disorder/Food crawl	A large amount of food/1.5 L of undigested food	Diffuse abdominal pain, nausea, and constipation Worsening symptoms and peritoneal signs	Nasogastric tube Partial gastrectomy
10	Wiedbrauck (8)	2022	F	23	Anorexia nervosa	Large amounts of food	Severe abdominal pain, nausea, and inability to vomit	Nasogastric tube Endoscopic treatment
11	Pitre (9)	2021	F	21	Anorexia nervosa, binge/purge subtype	Binge eating	Mild abdominal pain, constipation and abdominal distention	Nasogastric tube Esophagogastroduodenoscopy
12	Roa (21)	2021	F	43	Anorexia nervosa	3 L of semisolid output	Diffuse abdominal pain, and distension	Nasogastric tube
13	Achamrah (18)	2020	F	37	Bulimia nervosa	Binge eating/6.5 L of aspiration	Abdominal pain, distended abdomen with defense Acute renal failure	Nasogastric tube Total gastrectomy and jejunostomy
14	Craven (10)	2020	F	17	Anorexia nervosa of the restrictive subtype with occasional episodes of binge eating and purging	An entire family size pizza	Severe abdominal pain, retching, inability to vomit and obstipation Abdominal distension, tenderness and guarding Pneumatosis of the stomach wall	Oesophago-gastroscopy Gastrotomy
15	Nam (22)	2019	F	33	Wedding preparation-related stress	Heavy meals	Severe epigastric pain Diffuse intrahepatic portal vein gas	Gastric lavage Esophagogastroduodenoscopy
16	Dumouchel (15)	2017	F	26	Anorexia nervosa	Food and beverage, 1.5 L of water	Diffuse abdominal pain, distended with an extensive mass from the pelvis to the ribs Acute renal failure	Nasogastric aspiration
17	Foran (20)	2016	F	32	Binge/purge subtype eating disorder	A large amount of carbohydrates	Abdominal pain, abdominal distension and vomiting Extensive pneumatosis involving the stomach and portal venous gas	Emergency laparotomy
18	Usui (19)	2016	F	60	Anorexia nervosa	2 loaves of bread, 3 sweet buns, and 2 packs of instant noodles and 4.3 L of carbonated water and 1.4 L of low-malt beer	Severe abdominal pain, vomiting Unconscious and collapsed immediately after laying on the examination table in a supine position Subcutaneous emphysema	Died 30 min after admission to the hospital
19	Dewangan (23)	2016	M	17	Fast due to some religious reasons	Heavy meals/5 L of free fluid and undigested food	Severe pain and distension of abdomen Generalized tenderness and guarding of abdomen, pneumoperitoneum	Nasogastric tube Exploratory laparotomy
20	Dincel (24)	2016	F	24	No	Heavy meal, grapes and pomegranates/5 L of undigested food	Abdominal pain, nausea and vomiting Abdominal swelling, rigidity, and diffuse tenderness with peritonitis Anuria, cyanotic condition, no pulse of lower extremities	Nasogastric tube Laparotomy
21	Youm (25)	2015	F	21	Anorexia nervosa and bulimia nervosa	Heavy meal	Severe abdominal pain and distension, nausea and retching Anuria, cyanosis and no pulse in both lower extremities Pneumoperitoneum	Levin tube Exploratory laparotomy
22	Elsharif (26)	2014	F	18	Bulimia	Binge episodes/15 L of gastric content	Severe abdominal pain and distension Unresponsive, extensive mottling of the skin from the waist down, engorged neck veins and absent femoral pulses bilaterally	Emergency laparotomy and gastrotomy
23	Lemke (27)	2014	F	28	Anorectic behavior with starvation und excessive exercising	Binge eatings caused by conflicts at her workplace	Progressive abdominal discomfort and pain, distended and meteoritic abdomen with signs of peritonism	Nasogastric tube Gastrotomy
24	Franco-López (28)	2012	F	31	Bulimia nervosa	Massive food intake	Abdominal distention Hemodynamic instability and oliguria	Gastrostomy
25	Kim (29)	2011	F	26	Anorexia nervosa and bulimia nervosa	300 g of pork, 1 packet of ramen, 80 slices of bread, 1.5 L of Coke, and 1.5 liters of another soda	Severe abdominal pain, distended abdomen, epigastric tenderness, and hypoactive bowel sounds	Nasogastric tube
26	Tweed-Kent (30)	2010	F	26	Anorexia nervosa binge/purge subtype	Four beers and eating a Cobb salad	Diffuse abdominal pain, nausea, and an inability to vomit, distended, firm, and diffusely tender to palpation without peritonitis	Nasogastric tube Gastrotomy
27	Kim (31)	2009	F	34	Eating disorders	Binge eating	Extreme abdominal pain and dyspnea, marked abdominal distension with generalized muscle guarding	Gastric lavage Oesophagogastroduodenoscopy
28	Bravender (12)	2007	F	21	Bulimia nervosa	A variety of sweets, 1 pound of lemon bars	Severe, sharp, constant abdominal pain, severe nausea, faint bowel sounds; the abdomen was firm, distended, and the patient noted mild diffuse tenderness	Nasogastric tube
29	Gyurkovics (32)	2006	F	22	No	An enormous food intake	Diarrhea, vomiting and abdominal pain, distended abdomen The mesenteric and femoral pulses reappeared	Gastrotomy Nasogastric tube
30	Barada (33)	2006	F	24	Situational anxiety and depressive symptoms	Chicken and rice, bread, yogurt, biscuits, vegetables, and fruits	Diffuse abdominal pain radiating to the back, bloating, nausea, and an inability to vomit The abdomen was distended, tympanitic, and diffusely tender. Bowel sounds were hypoactive.	Nasogastric tube Gastrointestinal endoscopy
31	Luncă (34)	2005	M	22	Borderline mentally retarded	Binge eating episode	Acute-onset abdominal pain and progressively distended abdomen, nausea	Upper endoscopy
32	Holtkamp (35)	2002	F	16	Obesity and atypical anorexia nervosa	Four spring rolls, 1 kg of fruit, a portion of French fries, 200 g meat salad and 1.5 L water	Severe abdominal pain, a tender and bloated abdomen with poor bowel peristaltic Unsuccessful attempts to vomit and increasing pain	Laparotomy
33	Willeke (36)	1996	F	19	Anorexia nervosa	Bulimic binge	Abdominal pain, diffuse abdominal tenderness on pressure Sepsis, purulent intraabdominal fluid	Gastric tube under gastroscopic control Laparotomy
34	Saul (37)	1981	F	22	Anorexia nervosa	8 L of fluid and undigested food	Abdominal pain, constipation and an inability to vomit Femoral pulses were absent, and the lower extremities were cold and cyanotic	Nasogastric tube Gastrectomy

The pathophysiology of AGD due to binge-eating has not been fully elucidated (2, 3). Upon ingestion of a normal meal in healthy individuals, the proximal stomach undergoes receptive relaxation, thereby augmenting gastric volume to accommodate the ingested contents without elevating intragastric pressure (12). The core pathophysiological mechanism of AGD involves a sudden elevation in intragastric pressure. Critically, the severity of complications is not primarily determined by the volume or composition of ingested material, but rather by the degree of intragastric pressure elevation. Although the majority of reports on AGD associated with binge-eating predominantly emphasize excessive solid food ingestion, Zeng et al. presented a case of 40-year-old male with chronic dyspepsia developed gastric dilation following ingestion of 3.5 L of traditional Chinese liquid medicine over a 72-h period (2). Han et al. reported catastrophic gastric rupture following the ingestion of sodium bicarbonate after a large meal, where rapid gas production was identified as a key mechanism elevating intragastric pressure to the point of overt wall failure. This case demarcates the most severe end of the clinical spectrum, demonstrating that acute intraluminal pressure surges can directly overwhelm gastric integrity (5). In the present case, the same primary driver—extreme intragastric pressure from rapid gaseous distension due to massive carbonated beverage ingestion—precipitated a distinct complication. While this pressure may create gastric mucosal micro-defects, our radiological findings confirmed no visceral perforation (5, 7). Instead, the linking mechanism is consistent with the Macklin effect (4, 13). Here, the extreme gastric distension and the resultant forceful retching/vomiting acted synergistically. The former likely caused diaphragmatic displacement and transmitted pressure, while the latter constituted a powerful Valsalva maneuver. This combination generated the profound, acute rise in intrathoracic pressure necessary to induce alveolar rupture. Subsequently, air dissected along pulmonary interstitial sheaths toward the mediastinum (14). Thus, we propose a synthesized pathway for pneumomediastinum in AGD: the primary event is extreme intragastric pressure, which contributes to forceful retching, generating the intrathoracic pressure spike that triggers alveolar rupture (Macklin effect), with escaped air tracking centrally to cause pneumomediastinum. This alternative pathway of tissue failure, occurring without frank perforation, represents a serious but mechanistically different consequence within the same etiological framework of critical intragastric pressure elevation. Pneumomediastinum following AGD is rarely reported, and is a medical emergency.

The patient presented with significant laboratory abnormalities, primarily attributable to the severe physiological stress of AGD and its systemic consequences. The profound metabolic alkalosis (pH 7.61) is consistent with the loss of gastric acid due to protracted vomiting. The markedly elevated lactate level (9.1 mmol/L) reflects a combination of factors: hypoperfusion from significant fluid depletion (due to vomiting and inadequate intake), and potential tissue hypoxia secondary to increased intra-abdominal pressure from gastric distension, which may impede visceral perfusion. The leukocytosis (WBC 22.06 × 10^9^/L), along with elevated hemoglobin and platelet counts, is interpreted as a multifactorial response. The primary driver is likely a stress leukocytosis and hemoconcentration due to significant fluid loss. A secondary contribution from a potential low-grade inflammatory or infectious process related to gastrointestinal compromise cannot be entirely ruled out. The elevated creatinine and blood urea nitrogen are consistent with pre-renal acute kidney injury, again secondary to hypovolemia. In summary, the constellation of hematological and metabolic derangements is best understood as a systemic inflammatory and stress response initiated by the massive gastric distension, with significant contributions from hypovolemia due to vomiting and third-spacing of fluid.

The presence of abdominal complaints, such as persistent abdominal pain, distension, nausea and vomiting associated with binge-eating should alert emergency physicians to a diagnosis of AGD (15). Immediate gastric decompression is the mainstay of treatment (1). Nasogastric tube is the approach of choice (3). If drainage is ineffective and there is no evidence of perforation, endoscopic intervention can be employed to achieve decompression and identify the underlying etiology (8, 9, 16). When gastric dilation progresses despite conservative management and leads to signs of vascular compromise-manifests as gastric wall necrosis, abdominal compartment syndrome, acute renal failure, or lower limb ischemia, urgent surgical intervention is indicated (1, 5, 7, 10, 17, 18). Furthermore, the detection of extraluminal gas, such as free intraperitoneal air, pneumomediastinum, or portal venous gas, serves as a critical warning sign requiring emergency exploratory laparotomy (6, 19, 20). Therefore, clinicians need to recognize AGD as a true emergency, have a high index of suspicion when presented with such symptoms and signs, and upon diagnosis, perform immediate decompression and evaluation for surgery.

## Conclusion

This case highlights the necessity of early gastric decompression in binge-eating patients with significant carbonated beverage consumption who develop abdominal symptoms. The onset of hemodynamic instability or oliguria mandates urgent surgical intervention. Given the rarity of this condition, management must be individualized based on specific clinical and radiological findings.

## Data Availability

The original contributions presented in the study are included in the article/supplementary material, further inquiries can be directed to the corresponding author.
